# A Study of the Relationships between the Characteristics of the Village Population Structure and Rural Residential Solid Waste Collection Services: Evidence from China

**DOI:** 10.3390/ijerph15112352

**Published:** 2018-10-25

**Authors:** Sha Cao, Dingde Xu, Shaoquan Liu

**Affiliations:** 1 Institute of Mountain Hazards and Environment, Chinese Academy of Sciences, #9, Block 4, Renminnan Road, Chengdu 610041, China; caosha1993@126.com; 2 University of Chinese Academy of Sciences, No. 19A Yuquan Road, Beijing 100049, China; 3 Sichuan Center for Rural Development Research, College of Management, Sichuan Agricultural University, 211 Huimin Road, Wenjiang District, Chengdu 611130, China; dingdexu@126.com

**Keywords:** rural residential solid waste collection, supply and quality, village population structure, China

## Abstract

Based on survey data collected in 2016 from 100 villages across five provinces in China, this paper uses binary logistic model to explore the main factors related to the supply and quality of rural residential solid waste collection (RRSWC) services, especially focusing on the role of village population structure characteristics. It is indicated from the results that village population structure characteristics are significantly correlated with RRSWC services; specifically: (1) the total registered population of village households is significantly positively correlated with the supply of RRSWC services, however, the correlation between the registered population and the quality of RRSWC services is negative and the more the labors working and living outside the village, the less likely the village is to provide RRSWC services; (2) the villagers’ educational levels and RRSWC services show no significant correlativity at the present stage; (3) the preferential policies brought by population structure advantage play a positive role in the supply of RRSWC services but is not clearly related to the quality of RRSWC services, especially in the villages with a larger minority population. In addition, the study finds that, the economic development level of a village and the characteristics of village leaders are also positively correlated with the supply and quality of RRSWC services and; the natural and traffic conditions have no significant correlation with RRSWC services. This study can act as a reference for the further promotion of the development of RRSWC services in China.

## 1. Introduction

Residential solid waste (RSW) has become a worldwide problem, especially in developing countries and their rural areas. In 2012, the total amount of RSW generated in all cities globally reached 1.3 billion tons [1] and RSW emissions in developing countries also increased due to rapid economic growth and improved living standards. It has been predicted that the generation rates of RSW in developing countries will more than double over the next 20 years [1]. Unfortunately, compared with developed countries, the disposal technology and management systems for RSW in developing countries are not mature [2], with concern related to how to improve the coverage of management services [3], especially in rural areas. Affected by policy imbalances and the ability and willingness to pay, RSW management services in rural areas in developing countries are much less valued and accessible than those in urban areas [4,5,6]. Thus, RSW has become the main cause of pollution in the rural environment [7], which is often irreversible and threatens the health of residents [8,9].

Rural areas in China, which is the largest developing country in the world, account for 90% of its mainland; therefore, particular attention must be paid to the management of Chinese rural RSW (RRSW). Several studies have shown a significant inverted “U” curve relationship between daily per capita quantity of RRSW and net per capita annual income of farmers [10,11]. At present, China’s RRSW emissions are at a stage of rapid growth [12]. In 2010, the total annual quantity of RSW from rural areas in China reached approximately 234 million kg per year, exceeding the quantity of municipal RSW (157 million kg/year) for the first time [10], meaning that the annual growth rate of RRSW is estimated to be approximately 8–10% [13]. Traditionally, most RRSW in China was recycled onto agricultural land as organic fertilizer and the environmental pollution from RRSW was not serious [14]; however, the issue of RRSW is becoming increasingly complex and harmful [15]. Moreover, the unusual phenomenon of “transplanting big trees into cities and delivering waste to the countryside” in the rectification of urban environments in China transforms the rural areas into the cities’ natural garbage dumps, meaning that trends towards the “ruralization” of urban waste, as well as the “integration” of urban and rural waste, have become the norm [8]. Regrettably, however, due to a valuing of economic development over environmental protection, “focusing on cities instead of rural areas” and other historical reasons [5] (as in most developing countries [16,17,18]), RRSW has not been seriously valued in China for a long time [19], nor is the management situation currently very desirable [20]. According to the survey [21], RRSW management services are still non-existent amongst the majority of the 600,000 villages in China, which also includes the absence of specialized management and maintenance staff. Thus, RRSW treatment methods in China’s vast areas generally include unregulated dumping, burning waste in open sites and simple composting [22]. Through these treatment methods, the recovery rate of RRSW is very low and this have been likely to result in serious levels of pollution. Statistics show that, in China, 49.5% of environmental pollution in rural areas is caused by RSW [23].

However, very few studies have examined RSW in the rural areas of developing countries. Similar to the relative lack of RSW management in rural areas, the research on RSW management is still more concentrated in cities [24,25,26,27,28,29,30] and relatively little research has been done on RSW management in rural areas. First, there is a lack of quantitative research. Most international discussions on RRSW management focus on analyzing the emission composition and management status of RRSW, as well as on how to improve the management of RRSW from a technical and policy point of view [31,32,33]. Furthermore, the majority of studies that have been conducted in China are qualitative analyses of RRSW emission characteristics, management systems, laws and regulations [23,34,35,36,37,38]. The second issue is the lack of village-level research. Much of the existing quantitative research on this issue has explored the determinants of individual villagers or households on participating in RRSW management, such as the research on Thailand by Janmaimool et al. [39] and the research on South Africa by Oyekale et al. [40] but few have been conducted at the village level. The main reason for this is the difficulty in obtaining RRSW data at the village level. Thus, the third deficiency is the lack of data available to be used in research. Several Chinese scholars have explored the influencing factors of RRSW management supply at the village level [13,20,41,42,43]; however, their research is often based on small-scale survey data that is not sufficiently generalizable [13,20,23] or the data is outdated [41,43] and thus fails to reflect the latest developments in RRSW management in China. Finally, there is poor understanding around the stage of RRSW management, which leads to research on RRSW management services as a whole [13,42,43].

In fact, it was unanimously recognized that perfect RRSW management services generally consist of three parts: waste collection (including waste collection facilities and waste collection workers), waste transportation (the waste is transported to centralized treatment plants) and waste treatment (including classification, decomposition and other centralized scientific treatment, as well as emergency burial, burning and other non-scientific treatment) [11,42,44,45]. Given this, the management of RRSW cannot simply be seen as a whole. Whilst rural residential solid waste collection (RRSWC) services are the basic link in RRSW management, without the input of RRSWC there are no subsequent links to RRSW transportation and treatment. However, there is an understanding that the attention paid to RRSWC is inadequate. Lima’s [33] survey on Quilombola in Brazil found that only 30% of rural communities provide RRSWC services, less than 50% of Romanian villages provide RRSWC services in poor areas [46] and the differences in shares of villages with RSWC services across provinces are statistically significant in China [42]. However, apart from the study by Wang [44], few studies have further explored the factors that affect the supply of RSWC in villages. In addition, the quality of services in villages in China that have RSWC varies greatly [47,48]. Based on international experiences of RSWC, it has been shown that the quality of RSWC services affects the subsequent link to waste treatment, meaning that high-quality RSWC services can result in the improvement of treatment efficiency and the recovery rate of waste, as well as a reduction in waste treatment costs [2,49,50,51]. This notwithstanding, existing research invariably ignores the quality of RRSWC services. Therefore, it is necessary to gain a more in-depth understanding of the main factors related to the quality of RRSWC at the village level based on factors that relate to RRSWC supply.

In addition, the distinct role of villagers in RRSWC should not be understated. Previous studies have predominantly focused on the impact of social and economic conditions in RRSW management services but all RRSW management policies, regulations and public investment carried out in rural areas are aimed at improving the living environments of rural residents, as residents should be the focus. The population economist believes that people are the main body of social and economic activities and the population structure will react to social and economic development; therefore, different population structure characteristics between villages will inevitably impact the development of local RRSWC services in specific ways. Gaining a better understanding of the correlations between village population structure and RRSWC services can effectively help to predict the development trends of villages’ RSWC services under different population structure characteristics, thus helping to formulate effective development policies.

Guided by the abovementioned research and using China as a representative case among developing countries (i.e., nation-wide data collected on China in 2016), the current study focused on RRSWC services, which provide a fundamental link to RRSW management and the reaction of the population structure in population economics was used as the theoretical background. Thus, the current study explored the main factors that related to the supply and quality of RRSWC services at the village level, with a particular focus on the role of village population structure characteristics. This focus in turn could help to provide a reference point for decision makers with regard to the further provision of RRSWC services according to local conditions, as well as improving the quality of services. The key question this study posed was, ‘What are the quantitative correlations between the different demographic characteristics of villages and the supply and quality of RRSWC?’

## 2. Data and Methods

### 2.1. Research Area and Data Sources

**Figure 1:**
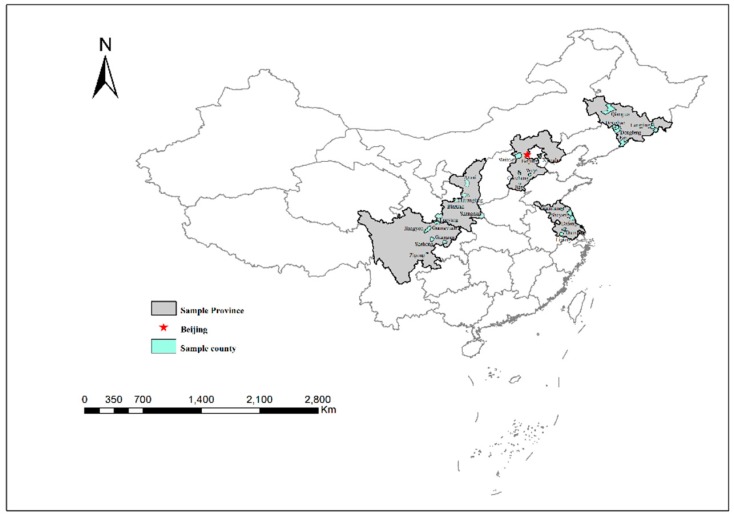
Location of the sample provinces and counties.

The data used in this paper are from an almost nationally representative survey in rural China that was conducted in 2016 by the Center for Chinese Agricultural Policy (data for 2015). We surveyed 100 villages from 50 townships in 25 counties located in five provinces (as a return visit project, our sample villages should have totaled 101 but two of the original villages in Jiangsu had merged before 2015; Figure 1). A stratified random sampling method was adopted and the village was taken as the final sample level. The sample selection process was as follows: firstly, according to the social and economic development level and agricultural production conditions, all provinces in China were divided into five major zones and one province was randomly selected from each major zone, leaving five provinces in total (Jilin, Jiangsu, Hebei, Shaanxi, Sichuan). Secondly, all counties in each sample province were presented in descending order by survey teams according to per capita Gross Value of Industrial Output (GVIO) (GVIO was used based on the study from Rozelle [52], which shows that it is one of the best predictors of both living standard and development potential and is often more reliable than net rural per capita income.) and divided into five equivalent groups. One county was randomly selected from each group, leaving a total of 25 counties. Finally, the townships in all the sample counties were divided into two groups in accordance with GVIO (one group with the per capita GVIO above the county median and one group with the per capita GVIO below the county median); and one township was randomly selected from each group, leaving 50 townships in total. The same method was adopted to select the final 101 sample villages (two sample villages in Jilin Province were merged and separated in the first round of the survey and, therefore, the information of the two villages was collected together, which continued in the subsequent surveys). The distribution of the sample area is shown in Figure 1. Among the five survey provinces, Sichuan contained the most extensive rural area and, at the same time, by the end of 2015, the proportion of rural population was the largest (52.31%), followed by Hebei (48.67%), Shaanxi (46.08%), Jilin (44.69%) and Jiangsu (33.48%) [53].

The research team comprised 5 teachers and more than 100 graduate students. Before the formal investigation, all investigators received rigorous training and pre-investigation to ensure the authenticity and reliability of the survey data. Next, the interviewees of the sample villages were interviewed face-to-face using the pre-prepared questionnaire. In this paper, the questions about a village’s RSW were all answered by the village head (because in China’s rural areas the village head has a comprehensive grasp of the basic situation of the village and is also an authority figure). When the village head was unable to participate in the interview, we turned to the village party branch secretary (because in rural areas the village party branch secretary and village head jointly manage village affairs and the secretary is seen as equal in authority to the village head). The main issues investigated in this study included whether the village provides RSWC services (“Were there residential waste collection services in your village in 2015?”) and the quality of a village’s RSWC services (“How about the quality of residential waste collection services in your village in 2015?”). We also collected data on seven structure characteristics of the villages’ populations, including the total registered population of the village households, minority populations, the number of migrant workers still living in the village, the number of migrant workers living outside the village, the number of illiterate individuals among the labor force, the number of college (college and above) students in the village in the past four years and the number of upper-level government staff from the village. The following data were also collected: (1) the economic development level of the village (the number of self-employed industrial and commercial households in the village and villagers’ per capita annual incomes); (2) village leaders’ characteristics (whether the village head is a new leader and the educational level of the village head); and (3) the natural and traffic conditions of the village (the type of cement or asphalt road nearest to the village and the terrain of the village).

### 2.2. Research Hypotheses and Variable Selections

**Figure 2:**
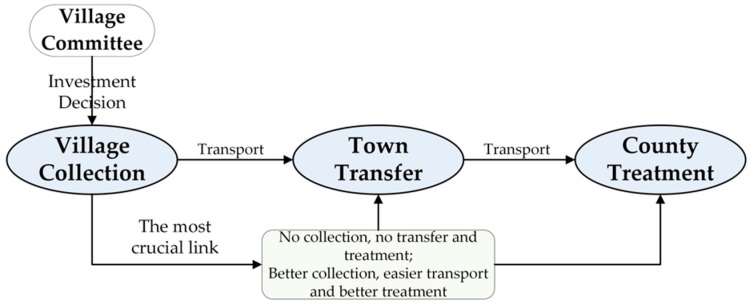
The management framework of RRSW in China.

“Village Collection, Town Transfer, County Treatment” [40,43] is currently the most common and efficient RRSW management model in China and the village committee is the investment decision maker for RRSWC services (Figure 2). However, the final executors and service objects are the villagers. As discussed previously, population structure characteristics can significantly react to social and economic development, therefore, when a village committee carries out investment decisions regarding RRSWC services, the role of the population structure characteristics of a village should not be overlooked. Firstly, according to Ye’s [41] research, the villagers’ demand is the most important driving force behind the supply of RRSWC services. Therefore, the characteristics of a village’s population size should be carefully considered, as, to a large extent, the population size of the village is related to the villagers’ demand for RRSWC services [36]. In general, the greater the population of the village, the more RRSW is produced, the more destructive it is on the landscape and environment if proper collection is not available [54] and the easier it is to attract the attention of villagers and stimulate the demand for better RRSWC services [36]. Additionally, the length of time that villagers live in a village may also be related to demand [20]. It must be noted that, in rural areas of China, there is a common phenomenon that numerous laborers go out to work. On the one hand, if these villagers live in a village for a shorter period of time, their demand for RRSWC services and investment willingness may be less intense [10,20]. On the other hand, these individuals may still return home after finishing their work and with their increased income and capacity to earn, they may be more willing to contribute to the improvement of their living environment, both for themselves and their families, which in turn will increase their demand and willingness to pay for RRSWC services [41]. Secondly, the villagers’ awareness of RRSW hazards and environmental protection should be considered. Moreover, as Kocasoy [55] has demonstrated, the education level of villagers plays a positive role in villagers’ awareness of the importance of RSW management, improving the education levels of villagers in developing countries can promote the efficiency of RRSW management. In addition, the studies conducted by many other researchers have shown that villagers with a higher level of education are more likely to recognize the importance of environmental protection and participate in RRSW management, especially in classified waste collection [2,6,40,45,47,56]. In rural China, the labor force is the most affordable group of RRSWC services in villages and their willingness to pay plays an important role in RRSWC services that are available in villages [41]. Therefore, their recognition of the importance of RRSWC is particularly important. Furthermore, according to Xu’s findings [57], due to the effects of social networks, villagers tend to display homogenization in terms of awareness towards RSW hazards and payment willingness for RRSWC services. As university students benefit from a higher level of education and urban life experience, they may have more advanced environmental protection ideas than those of other villagers. Besides this, they may encourage their own families to support RRSWC and thus the willingness of villagers around them to pay for services may be promoted as well. Finally, it is also necessary to consider possible preferential policies and investment for a village from the upper-level governments [41], which result from the structure characteristics of a village’s population. In China, as a result of ‘Policy towards Nationalities’ [58], if there is a higher minority population in a village, that village would be more likely to be obtaining preferential policies and public service investments from the upper-level governments [44]. Besides this, because of the existence and development of informal connections (guanxi) in rural China [59], villagers working in upper-level governmental roles would be more likely to favor their home village when allocating resources, such as public goods investment [13].

Based on the theoretical analysis outlined above, this paper considers whether the village provides RRSWC services and the quality of these RRSWC services as the dependent variables. Moreover, the following are classified as variables of particular interest: the total registered population of village households, the proportion of migrant workers living in a village in the labor force and the proportion of migrant workers living outside in the labor force are used to characterize villagers’ demand for RRSWC services; the proportion of illiterate individuals in the labor force and the number of college (college and above) students in a village in the past four years are used to characterize villagers’ awareness of RRSWC services; and, finally, the proportion of a minority population and number of civil servants in upper-level governmental roles in a village’s total population are used to characterize a villagers’ political advantages in RRSWC services from the upper-level government. In addition, to minimize the influence of missing variables on the variables of interest (with reference to the existing empirical results [13,14,22,43,44,60]), indicators reflecting the level of economic development of a village, the characteristics of village leaders and the natural and traffic conditions were added as control variables (Table 1).

Moreover, the current study proposed the following hypotheses on possible links between the structure characteristics of a village’s population and the supply and quality of RRSWC services: (1) H1: the demand of villagers will be positively related to the supply and quality of village RSWC services (i.e., the greater the demand of the villagers, the greater the pressure on the village committee and the more likely it is to provide better services); (2) H2: residents’ level of awareness of RRSW will be positively related to the supply and quality of village RSWC services (i.e., residents with a greater awareness of RSW hazards will be more likely to participate in RSW management and pay for RSWC services); and (3) H3: policy inclination brought by population structure will be positively related to the supply and quality of village RSWC services (i.e., the greater the policy inclination from the upper levels of government, the more likely the village is to obtain further RSWC investment).

### 2.3. Models and Methods

As the dependent variable “whether the village provides RSWC services” may only give a “yes” or “no” result (the discrete results of a binary variable), the use of a probability model is ideal. In the initial survey, the quality of village RSWC services—the second dependent variable—was divided into three grades, namely “good,” “general” and “poor.” In the actual process of the data collection, it was found that among the 75 sample villages providing RRSWC services, the quality of RRSWC services was “poor” in only three villages and, before the analysis of the binary logistic model of RRSWC service quality, the author used the ordered multi-classified logistic model to conduct the analysis. However, it was found that the model did not pass the parallel tests and the dependent variables did not conform to the treatment condition of the ordered multi-classified variables. Thus, in order to achieve the reliability and comparability of the model results, “general” and “poor” were merged by the author and regarded as another binary variable for this study. The binary logistic model was used to estimate the binary response probability based on one or more predictor variables (or independent variables), which is an effective model for the multiple regression analysis of the dependent variable as a binary variable. Hence, this paper predominantly uses the binary logistic model to conduct the analyses.

The general form of the binary logistic model is as follows:

$$\log itP=\ln\left(\frac{p}{1-p}\right)=\beta_{0}+\sum_{i=1}^{n}\beta_{i}X_{i}$$

where, logit *P* indicates the probability of the village providing waste collection services or good service quality; $\beta_{0}$ is the constant term; n denotes the number of influencing factors; $\beta_{i}$ refers to the regression coefficient for the influencing factor *i*; and $X_{i}$ means the sample value of the influencing factor *i*.

Before the analysis of the regression model was conducted, “whether the village provides RSWC services” and “the quality of RSWC services” were taken as the dependent variables to carry out the multi-collinearity tests in order to avoid explaining how multi-collinearity between variables affected the accuracy of the model. The two test results showed significant collinearity (the correlation coefficient was greater than 0.8) between the variable *In* (the proportion of migrant workers living in the village in the labor force) and other variables, so the variable *In* was removed. The variables used in the final model are given in Table 2.

## 3. Results

### 3.1. The Descriptive Statistics Analysis

**Figure 3:**
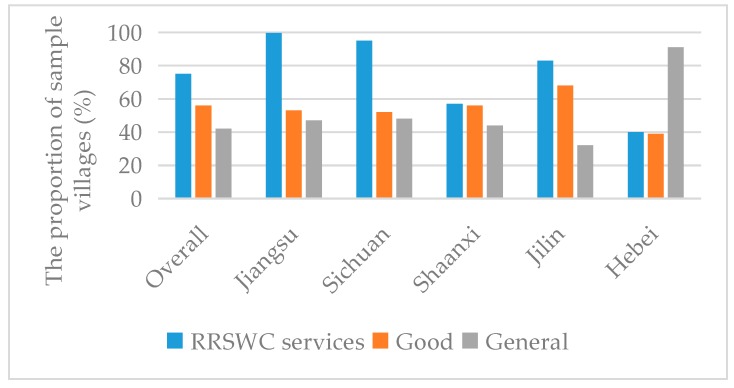
The descriptive statistics analysis results of the variables involved in the model.

Table 2 shows the descriptive statistics of the variables involved in the model. As shown in Table 2, among 100 sample villages in China, 75 villages provided RRSWC services, accounting for 75% of the survey sample and the average level of RRSWC service quality is 0.55. In the five sample provinces, RSWC services were provided in all sample villages in Jiangsu Province and the proportion of villages providing RRSWC services was the largest, followed by Sichuan, Jilin and Shaanxi. In the sample provinces, the proportion of RRSWC services provided in Hebei was the smallest, with only 40% of villages providing the services. In all the samples of villages providing RSWC services, the proportion of “good” quality RRSWC services provided was largest in Jilin and, overall, the service quality in Shaanxi and Hebei was relatively poor (Figure 3).

Moreover, the average resident population of the sample villages was 1759 and 32.16% of the labor force worked and lived outside. As for the education level of the villagers, 4.21% of the labor force were illiterate and the average number of college (college and above) students in the past four years was approximately 41. Furthermore, the proportion of the minority population was 5.47% and every sample village had approximately 17 persons working in upper-level government.

### 3.2. Econometric Model Results

Table 3 presents the econometric model results of the supply and quality of RRSWC services. Among them, Model 1 and Model 2 are the model results of the relationship between the independent variables and the supply of RRSWC services and Model 3 and Model 4 are the model results of the relationship between the independent variables and the quality of RRSWC services. Moreover, in order to test the robustness of the focus variables’ influence on RRSWC services in the models, Model 1 and Model 3 only include focus variables, while Model 2 and Model 4 include both focus and control variables. From the overall test statistics of models 1–4, it is apparent that, overall, the degree of fit of all the models is good and, according to the test statistics of the models (Wald chi^2^), it is suggested that Model 1 and Model 2 are significant at the 0.01 level, while Model 3 is significant at the 0.1 level and Model 4 is significant at the 0.05 level, indicating that at least one of the independent variables has a significant relationship with the dependent variable.

#### 3.2.1. Factors Related to the Supply of RRSWC Services

From Model 1 and Model 2 it can be seen that the results of the focus variables in this study are robust; therefore, in further analyses, the results of Model 2 are adopted to explain the main factors that relate to the supply of rural RRSWC services.

Using the results of Model 2, it is apparent that the correlations between each focus variable of the village population structure characteristics and whether the village provides RRSWC services are mostly consistent with the research hypotheses. (1) The three variables—*Population, Student* and *Servant*—are all positively correlated with the supply of RRSWC services. Among them, *Population* and *Servant* have a significance level of 0.01 and the Exp(B) values (OR) are 1.002 and 1.065, respectively, indicating that villages with a larger population and a greater number of upper-level government staff are more likely to be providing RRSWC services than others. Moreover, the positive correlation between *Servant* and RRSWC services supply is greater than it is between *Population* and RRSWC services supply, suggesting that the human resource advantage of the village population is an important driving force in the promotion of RRSWC services supply. On the other hand, the correlation between *Student* and the supply of RRSWC services is positive but not significant. (2) The variables *Out* and *Illiteracy* are negatively correlated with the supply of RRSWC services. In addition, *Out* is significant at the 0.05 level and the Exp(B) value (OR) is 0.972, while the correlation between *Illiteracy* and the supply of RRSWC services is not significant. (3) In the research hypotheses, the correlation between *Minority* and the supply of RRSWC services is positive but the model results show that *Minority* is negatively correlated with the supply of RRSWC services, although this is not significant.

In terms of control variables, *Income* (logarithms) and *Appointment* are significantly positively correlated to the supply of RRSWC services and, specifically, the richer villages with new leaders are more likely to be providing RRSWC services. The correlations between the remaining control variables (*Commerce*, *Education*, *Road*, *Terrain*) and the supply of RRSWC services are positive but not significant.

#### 3.2.2. Factors Related to the Quality of RRSWC Services

The results of Model 3 and Model 4 indicate that the focus variables in this study are robust; therefore, in further analyses, the results of Model 4 are adopted to explain the main factors that relate to the quality of RRSWC services.

Model 4 shows the econometric model’s results of a correlation between all the independent variables and the quality of RRSWC services. The variable *Servant* is not included in the village population structure characteristics because the analyses indicated that the correlation coefficient of this variable and the dependent variable is 0, meaning there is no correlation between them; therefore, this variable was removed from the model and, following this removal, the overall degree of model fit improved.

From the results of Model 4, it is apparent that among the characteristics of village population structure, only *Student* is positively correlated with the quality of RRSWC services, although this is not a significant relationship. *Population*, *Out*, *Illiteracy* and *Minority* are all negatively related to the quality of RRSWC services, wherein the relationship between *Population*/*Minority* and the quality of RRSWC services was remarkable. Specifically, villages with a larger population and minority population were more likely to be providing lower-quality RRSWC services and a larger proportion of migrant workers living outside in the labor force and the larger proportion of illiterate individuals in the labor force can reduce the quality of RRSWC services. These relationships, however, were found not to be significant.

The two variables representing a village’s economic development characteristics (*Commerce* and *Income*) were both significantly positively correlated with the quality of RRSWC services, indicating that level of economic development is an important factor that relates to the quality of RSWC services in rural China at present. Among village leaders’ characteristics, the correlation between *Appointment* and the quality of RRSWC services is positive but not significant, while *Educational* was significantly positively correlated with the quality of RRSWC services. The model results for the natural and traffic conditions are consistent with the research hypotheses and road level (*Road*) and the terrain (i.e., flatness) of a village (*Terrain*) are positively related to the quality of RRSWC services but are not significant.

## 4. Discussion

The object of this study was to research RRSWC services in China, which are the basic link to RRSW management. At the village level, this study focuses on whether the village provides RRSWC services, as well as the quality of RRSWC services provided and discusses the main factors that related to the supply and quality of these RRSWC services, paying especially close attention to the role of village population structure characteristics.

Research results from the present study illustrated that: (1) The correlations between villagers’ demand (population size and length of residence) and the supply of RRSWC services were consistent with H1 and the majority of the research results [14,36,40,43,52]. Villagers’ demand was found to be an important factor promoting RRSWC service supply. *Population* showed a significantly positive relationship to the supply of RRSWC services because if the population is large enough to achieve good economic returns and thus reduce costs [57], then the village committee would be more likely to provide RRSWC services. However, the relationship between *Population* and the quality of RRSWC services was significantly negative. This may be because the current supply of RRSWC services was still insufficient relative to demand in China [44]; therefore, it is possible the service quality is reduced in the densely populated villages owing to a lack of adequate supply. Moreover, *Out* was negatively related to both the supply and quality of RRSWC services, which may be because the labor migration reduced the permanent population in the village and, thus, lowered the demand for RRSWC. Conversely, these villagers were not long-term residents and thus improvement to the hygienic environment in the village would have little influence on the improvement of their welfare relative to long-term residents and, therefore, they might not be concerned with RRSWC [20]. (2) There was a discrepancy with H2. The correlation between the recognition level (education level) of villagers regarding RRSWC services and the supply and quality of RRSWC services was positive; however, the correlation was not significant. This is understandable, although highly educated villagers generally have higher requirements for rural sanitation [20]. Ye’s survey [36] showed that, in rural China, the villagers generally believed the provision of RSWC was the responsibility of the village committee, not themselves and they had not formed the concept of environmental protection or internalized RRSWC into spontaneous behavior. (3) Unexpectedly, *Minority* was negatively related to both the supply and quality of RRSWC services and the relationship to quality was significant. This was opposite to the research hypothesis in the present study but was consistent with Ye’s conclusion [41]. A reason for this may be that although the government will strengthen policy preference to minority areas, owing to a low economic development level in these areas and the unique ethnic culture and lifestyle of minorities, the importance of environmental protection is not fully understood; therefore, it is difficult to achieve the expected policy effect. Moreover, *Servant* was significantly positively related to the supply of RSWC services, which was consistent with the conclusions of H3, Pan [13] and Han [61]. However, the number of villagers working in upper-level governmental roles was not clearly related to the quality of RRSWC services, indicating that the current government may only be concerned about whether the service was provided, rather than the quality of the RSWC services. Thus, the implementation of policies must be strengthened.

Additionally, the relationships between village economic characteristics (especially *Income*) on RRSWC services were most significant regarding the abovementioned control variables and are consistent with the conclusions of Wang et al. [20,42,44]. The economic development level determined the ability to supply RRSWC services by villages and showed that richer villages can provide RRSWC services of a higher quality. The leadership characteristics of villages were also significantly related to RRSWC services. In China, there is a saying, “xin guan shang ren san ba huo,” which means “a new broom sweeps clean.” Specifically, new village leaders may tend to provide RRSWC services in order to show their political achievement, which has also been verified by the research results presented by Pan et al. [3,42]. Moreover, as Ye’s [36] results confirmed, highly educated leaders can often provide better RRSWC services because they are often more environmentally conscious. The natural and traffic conditions were not significantly related to RRSWC services, which diverges from Wang’s research results [20,42]. This result could be due to subsequent RRSW management (transportation and processing) being more dependent on natural and traffic conditions; whereas, RRSWC services are less influenced by these conditions.

Compared with the previous research, the contributions of the present study are as follows. First, this study uses China as the representative of developing countries and adopts the latest nationally representative data collected by investigation in 2016, making the conclusions more universal and time-sensitive. Second, using the village as the research scale, the present study quantitatively analyzed the main factors that relate to RRSWC services (which is the basic link to RRSW management) from the perspective of the structural characteristics of the village population and, in turn, extended the insufficient attention previously paid in existing research to RRSWC. Finally, by exploring the main factors that relate to RRSWC supply, the study further explored the main factors that directly correlated with the quality of RRSWC. This is a new attempt at RRSWC research, which provides inspiration for follow-up studies. Thus, despite the different socio-economic and cultural backgrounds of different countries and regions, this quantitative research (using the latest national data from China as the largest developing country globally) can provide a reference point for research on and the development of RRSWC in other developing countries worldwide.

## 5. Conclusions and Suggestions

From the perspective of the population structure characteristics at the village level, this study applies the data of 100 villages throughout five provinces in China in 2015 that was collected to explore the factors that relate to the supply and quality of RRSWC services, which is the basic link to RRSW management services in China.

The results clearly demonstrate that the different characteristics of a village’s population structure relate in different and significant ways to the supply and quality of RRSWC services. The main findings are as follows:

1. Villagers’ demand was an important factor that related to the supply and quality of RRSWC services. The relationship between total household registration and the supply of RRSWC services was found to be significantly positive; however, it played a significantly negative role in the quality of RRSWC services. The proportion of migrant workers living outside in the labor force was negatively correlated with the supply and quality of RRSWC services, although this relationship was not significant.
2. The relationships between the education level of villagers and the supply and quality of RRSWC services were not significant. The proportion of illiterate individuals in the labor force was negatively related to the supply and quality of RRSWC services, while the relationships between the number of college (college and above) students in the village in the past four years and the supply and quality of RRSWC services were positive. However, these relationships were not all significant.
3. The relationships between the political advantages resulting from the structure characteristics of the village population and the supply and quality of RRSWC services were inconsistent. The proportion of the minority population was negatively related to both the supply and quality of RRSWC services and the relationship to quality was significant. The number of villagers working in the upper-level governments was significantly positively correlated with the supply of RRSWC services but showed no relationship to the quality of RRSWC services.
4. In terms of control variables other than the characteristics of village population structure, the number of self-employed industrial and commercial households and villagers’ per capita annual incomes—which represented the level of village economic development—were both positively related to the supply and quality of RRSWC services. Secondly, the characteristics of village leaders were also significantly related to the supply and quality of RRSWC services. New village leaders tend to provide RRSWC services and highly educated leaders may be more likely to provide RRSWC services with higher quality. Finally, the relationships between the villages’ natural and traffic conditions and the supply and quality of RRSWC services were not significant.

Based on these findings, the following provides some important policy suggestions:

1. Villages with different population structures should take different measures to improve the level of RRSWC services. First, the supply of RRSWC services should be adjusted according to the village population—especially the resident population—in order to provide enough services to match the demand to ensure the RRSWC services are of a high quality. Second, it is important to publicize and promote a way of thinking that is guided by science and conscious of environmental sustainability and also to improve the utilization of investment and implementation of policies in minority areas.
2. In view of the significant relationships found between the level of village economic development and the supply and quality of RRSWC services, the capital investment on RRSWC services should be increased in areas where economic development is relatively low in order to relieve the burden on local village committees and residents. Besides this, the characteristics of village leaders should also be considered carefully, with more highly educated leaders nominated where possible. Moreover, not only the supply of RRSWC services but also further assessment of the service quality should be included in the assessment system of village leaders.

## Figures and Tables

**Table 1:** The definition and data description of the variables involving in the model ^1^.

Variable Type	Variables	Definition and Assignment	Mean	SD	Expected Direction
Dependent variable	Service	Whether the village provides RRSWC services (1 = yes; 0 = no)	0.75	0.44	
	Quality	The quality of RRSWC services (1 = good; 2 = general; 3 = poor)	1.49	0.58	
Village population structure	Population	The total registered population of the village households (measurement unit is person)	1758.84	1302.19	+
	In	The proportion of migrant workers living in the village in the labor force (%)	17.05	2.25	+
	Out	The proportion of migrant workers living outside in the labor force (%)	32.16	25.43	-
	Illiteracy	The proportion of illiterates in the labor force (%)	4.21	8.26	-
	Student	The number of college (college and above) students in the village in the past four years (measurement unit is person)	40.75	89.48	+
	Minority	The proportion of the minority population in the total village population (%)	5.47	17.01	+
	Servant	The number of upper-level government staff from the village (measurement unit is person)	16.59	31.13	+
Village economic development level	Commerce	Self-employed industrial and commercial households in village (measurement unit is household)	56.96	94.93	+
	Income	Villagers’ per capita annual income (logarithm) (yuan)	8.91	0.64	+
Village leaders	Appointment	Whether the village head is a new leader (elected or appointed in two years, 1 = yes; 0 = no)	0.19	0.39	+
	Education	Educational level of village leader (1 = primary; 2 = middle; 3 = high; 4 = College or above)	3.9	0.95	+
Village natural and traffic conditions	Road	The type of cement or asphalt road nearest to village (1 = national highway; 2 = provincial highway; 3 = county road; 4 = village road; 5 = others)	3.67	1.15	-
	Terrain	The terrain of village (1 = plain; 2 = hill; 3 = mountain)	1.84	0.9	-

^1^ Due to the larger variation of the original data of “villagers’ per capita annual income” in the variables, the logarithmic processing is carried out to make the model more stable; 1 dollar ≈ 6.727 yuan in 2015.

**Table 2:** The definition and data description of the final variables involving in the model ^1^.

Variable Type	Variables	Definition and Assignment	Mean	SD	Expected Direction
Dependent variable	Service	Whether the village provides RRSWC services (1 = yes; 0 = no)	0.75	0.44	
	Quality	The quality of RRSWC services (1 = good; 2 = general; 3 = poor)	1.49	0.58	
Village population structure	Population	The total registered population of the village households (measurement unit is person)	1758.84	1302.19	+
	Out	The proportion of migrant workers living outside in the labor force (%)	32.16	25.43	-
	Illiteracy	The proportion of illiterates in the labor force (%)	4.21	8.26	-
	Student	The number of college (college and above) students in the village in the past four years (measurement unit is person)	40.75	89.48	+
	Minority	The proportion of the minority population in the total village population (%)	5.47	17.01	+
	Servant	The number of upper-level government staff from the village (measurement unit is person)	16.59	31.13	+
Village economic development level	Commerce	Self-employed industrial and commercial households in village (measurement unit is household)	56.96	94.93	+
	Income	Villagers’ per capita annual income (logarithm) (yuan)	8.91	0.64	+
Village leaders	Appointment	Whether the village head is a new leader (elected or appointed in two years, 1 = yes; 0 = no)	0.19	0.39	+
	Education	Educational level of village leader (1 = primary; 2 = middle; 3 = high; 4 = College or above)	3.9	0.95	+
Village natural and traffic conditions	Road	The type of cement or asphalt road nearest to village (1 = national highway; 2 = provincial highway; 3 = county road; 4 = village road; 5 = others)	3.67	1.15	-
	Terrain	The terrain of village (1 = plain; 2 = hill; 3 = mountain)	1.84	0.9	-

^1^ Due to the larger variation of the original data of “villagers’ per capita annual income” in the variables, the logarithmic processing is carried out to make the model more stable; 1 dollar ≈ 6.727 yuan in 2015.

**Table 3:** Econometric model results of the village population structure and RRSWC services ^1^.

Variables	The Providing of RRSWC Services		The Quality of RRSWC Services	
	Model 1	Model 2	Model 3	Model 4
Population	0.002 ***	0.001 **	−0.003 *	−0.001 **
	(0.001)	(0.001)	(0.002)	(0.000)
Out	−0.028 **	−0.026 *	−0.007	−0.003
	(0.013)	(0.015)	(0.010)	(0.013)
Illiteracy	−0.027	−0.010	−0.011	−0.030
	(0.033)	(0.040)	(0.035)	(0.046)
Student	0.026	0.030	0.001	0.013
	(0.024)	(0.027)	(0.005)	(0.011)
Minority	−0.045	−0.023	−0.057	−0.070 *
	(0.029)	(0.028)	(0.036)	(0.040)
Servant	0.063 ***	0.077 ***		
	(0.021)	(0.026)		
Commerce		0.022		0.016 **
		(0.014)		(0.007)
Income		0.929 **		0.499 **
		(0.651)		(0.518)
Appointment		0.926 *		0.513
		(0.892)		(0.798)
Education		0.342		0.354 *
		(0.350)		(0.319)
Road		−0.244		−0.243
		(0.291)		(0.273)
Terrain		−0.250		−0.601
		(0.458)		(0.405)
Constant	−2.049 **	−14.478 **	−1.961	−5.759
	(0.822)	(6.270)	(0.708)	(5.344)
**Overall test statistics**				
Wald chi^2^(χ)	36.905 ***	46.155 ***	17.058 *	25.449 **
Cox & Snell *R*^2^	0.309	0.370	0.190	0.291
Nagelkerke *R*^2^	0.457	0.547	0.220	0.389
Hosmer-Lemeshow value	13.699 n.s.	6.250 n.s.	9.246 n.s.	4.124 n.s.

^1^ Robust standard errors in parentheses; *, ** and *** represent significant difference at: *p* = 0.10; *p* = 0.05 and *p* = 0.01; n.s. represent *p* > 0.05.
